# The prevalence of PTSD and coping strategies among Palestinian mental health professionals during political violence and wartime

**DOI:** 10.3389/fpsyt.2024.1396228

**Published:** 2024-06-07

**Authors:** Muna Ahmead, Mariam Abu Turki, Louy Fawadleh

**Affiliations:** Faculty of Public Health, Al Quds University, Jerusalem, Palestine

**Keywords:** war, coping strategies, PTSD, mental health professionals, Palestine

## Abstract

**Background:**

In times of war, mental health professionals are at an increased risk of developing psychological problems, including posttraumatic stress disorder (PTSD). The effects of conflicts or wars on mental health professionals in Palestine and their coping methods of dealing with these challenges remain unknown. This study aimed to assess the prevalence of PTSD symptoms and strategies for coping among mental health professionals in Palestine, in light of the ongoing Gaza war and political violence.

**Methods:**

The study utilized a cross-sectional research design. Self-reported questionnaires, including the PCL-5 and Brief COPE scales, were used to gather data. The relationship between the research variables and PTSD symptoms was investigated using frequencies, percentages, bivariate analysis, Pearson correlation, and Pearson’s chi-square test.

**Results:**

A total of 514 participants were recruited, with an estimated prevalence of PTSD of 38.7%. Furthermore, the multivariate analysis revealed that having a prior history of trauma and feeling disabled or unable to deal with your patients during the current Gaza war and Israeli–Palestinian political violence increases the likelihood of developing PTSD symptoms. In addition, using venting, self-blame, and behavioral disengagement as coping strategies increases the likelihood of developing symptoms of PTSD. Moreover, using acceptance and substance use as coping strategies reduces the risk of developing PTSD symptoms.

**Conclusion:**

The findings revealed a high prevalence of PTSD symptoms among mental health professionals during wartime and political violence. As a result, mental health professionals need immediate assistance in enhancing their mental wellbeing through supervision, psychotherapy, and comprehensive and continuous training.

## 1 Background

War has a significant impact on the mental health of the civilian population (1, 2). War destroys communities and families, and it frequently stifles a country’s social and economic development. Furthermore, it has long-term consequences, causing physical and psychological harm to the population (1, 2). According to the World Health Organization (WHO), approximately 10% of people who experience traumatic events during armed conflicts will develop serious mental health problems such as depression and anxiety (3). A lack of qualified mental health professionals and inadequate mental health services make it difficult to implement specialized mental health programs tailored to the needs of victims during political conflicts and wars (4).

Mental health professionals are thought to be vulnerable to displaying symptoms resembling posttraumatic stress disorder (PTSD) through their patients’ stories, even if they were not directly exposed to their clients’ traumatic events (5, 6). This phenomenon, known as vicarious traumatization, can happen when mental health professionals are overly sympathetic to clients who have been through trauma (7). As a result, when faced with obstacles that directly contradict the demands of their profession, mental health professionals may lose their own internal sense of distinction between their personal and professional identities (8–10). Cohen et al. revealed that one-third of the therapists surveyed believed that disclosing a traumatic experience would be detrimental to the therapist. Furthermore, 20% of the therapists reported that the patients’ traumatic materials triggered painful memories from their own lives, which they found intrusive and difficult. Furthermore, 11% of therapists expressed feelings of helplessness, anxiety, and vulnerability (11).

PTSD is a stress-related syndrome that many people experience after being exposed to severe or potentially fatal traumatic events. It is caused by a psychological inability to cope with and recover from trauma and has both physical and mental consequences (12). There has been little research published on how political violence and war affect the mental health of mental health professionals, particularly in terms of PTSD. Conflict-related mental health research primarily focuses on the general public (13). Veronese and Pepe, for example, conducted a longitudinal study to look into the cumulative network of associations between trauma-related symptoms, professional burnout, and psychological distress among healthcare practitioners working in emergency settings in Gaza and the West Bank. The findings revealed that trauma-related symptoms had a direct relation to professional burnout and psychological distress. However, trauma-related symptoms were more strongly associated with professional burnout than psychological distress (14).

Coping mechanisms have been linked to a variety of mental illnesses, including PTSD (15), which are defined as attempting to avoid or mitigate risk, damage, and loss while also reducing anxiety associated with them. Coping mechanisms include “problem-focused” and “emotion-focused” coping strategies (16–18). Recognizing effective coping techniques earlier in the traumatic event may be most important for preventing symptoms, whereas understanding these strategies for managing PTSD symptoms may be highly helpful in guiding treatment interventions (19).

Israel’s armed forces in Palestine have ruled over the West Bank and Gaza Strip since 1967. Four wars have broken out in the Gaza Strip in the last 13 years (20). On 7 October 2023, a new war between Israel and Palestine broke out in the Gaza Strip. Thousands of Palestinians have died or been injured (19). Furthermore, there has been a significant increase in Israeli military-related violence in the West Bank (21). Palestinians in the West Bank have suffered from psychological and social traumas in addition to physical traumas as a result of Israel’s border closure, military occupation, and the consequences, which include a collapsing economy, a lack of jobs, altered living conditions, a weak healthcare system, and a lack of hope for a better future (22). Social trauma is defined as incidents that involve severe rejection or humiliation and pose a significant social threat (23). Individuals may experience psychological distress as a result of forced migration, ethnic cleansing, genocide, mass killings, or military defeat (24). Social trauma occurs when the social system fails to fulfill its obligations, such as protecting, assisting, and showing compassion to those experiencing economic hardship, political instability, war, or poverty (25).

Palestine is facing a significant human resource shortage, particularly in the fields of psychiatry, psychology, social work, and mental health nursing. It is worth noting that there are only 38 licensed psychiatrists (26) and 17 nurses working in community mental health facilities for a population of 5.35 million Palestinians (27). Unfortunately, there is no official information available on the number of other mental health specialists, such as social workers, psychologists, counselors, and psychotherapists. According to unpublished data, 709 of them were officially licensed and registered with the Palestinian Ministry of Health (2003). Many psychologists, social workers, and counselors offer mental health services without having a graduate degree in psychotherapy. Despite these distressing incidents, little is known about how mental health professionals become vulnerable to mental illnesses during wartime when they should be caring for their patients and providing psychological and mental health services and interventions to victims and vulnerable individuals. According to Sharkansky et al., most research on the effects of coping during trauma is based on data collected months or years after the index event. This raises the question of whether current symptoms influence coping memory during stress (19). Therefore, this study aimed to assess the prevalence of PTSD and coping mechanisms among Palestinian mental health professionals during the current Gaza war and political violence. It also sought to investigate the link between PTSD, sociodemographic and psychological factors, and coping strategies. Finally, it sought to examine the factors that influence the onset of PTSD symptoms.

## 2 Materials and methods

### 2.1 Study design and sampling

The study was a descriptive cross-sectional survey that ran from 22 December 2023 to 1 January 2024. It targeted all Palestinian mental health professionals currently working in Palestine during the ongoing Gaza war and political violence, including psychologists, counselors, social workers, psychiatrists, mental health nurses, and academics specializing in mental health at Palestinian universities. Participants were chosen using convenience and snowball sampling methods. Data were gathered using an anonymous online self-administered survey. Participants were asked to complete an electronic version of the questionnaire, designed using Google Forms. The study link was distributed to participants via a variety of channels, including social media, WhatsApp, emails, and therapy-related organization websites. This was done in response to the Israeli military’s closure and movement restrictions in the West Bank and Jerusalem. Furthermore, participants were asked to share the link with mental health practitioners throughout the country, and 514 from Jerusalem and the West Bank responded.

### 2.2 Tools and measures

The study used a self-reported questionnaire, which had the following three sections:

#### 2.2.1 A sociodemographic and psychological history data sheet

It gathered data on the participants’ age, gender, marital status, employment status, education, place of residence, place of employment, years of experience, years of employment, and trauma and crisis training at their workplaces and educational institutions. Participants were also asked about psychological changes resulting from the ongoing Gaza war and Israeli–Palestinian political violence compared to the pre-war period, as well as any previous traumatic events. Additionally, they were also questioned about the number of patients they treated during the first 2 months of the current Gaza war and Israeli–Palestinian political violence, as well as whether they felt incapable of caring for their patients during the current conflict. Furthermore, they were asked whether the current Gaza war and Israeli–Palestinian political violence had a negative impact on their psychological state. Finally, they were asked if they needed training in crisis intervention and treating victims of war and political violence.

#### 2.2.2 Posttraumatic distress symptoms

This section included the PCL-5, a 20-item scale designed to assess PTSD symptoms using the DSM-5 PTSD symptom criteria. Respondents rate each item on a scale of 0 (“not at all”) to 4 (“extremely”) to indicate how much that specific symptom bothered them the previous month. The total score for the 20 items yields an overall symptom severity score (0–80). A PCL-5 cutoff score of 31 to 33 suggests the presence of likely PTSD in any sample (28). Cronbach’s alpha was 0.935.

#### 2.2.3 Coping

This section included the Brief COPE scale, developed by Carver in 1997 (29), consisting of 28 questions. Both cognitive and behavioral coping strategies are included, and respondents indicate whether they have used a coping response on a four-point Likert scale (1 = I have not been doing this at all; 2 = I have been doing this a little bit; 3 = I have been doing this an average amount; 4 = I have been doing this a lot), with higher scores representing more coping strategies used by respondents. The Brief COPE scale assesses the following coping mechanisms: self-distraction, active coping, denial, substance abuse, emotional and instrumental support, behavioral disengagement, venting, positive reframing, planning, humor, acceptance, religion, and self-blame. Cronbach’s alpha was 0.854. The questionnaire was translated into Arabic and then translated back into English. To ensure the accuracy and understandability of the Arabic terminology, it was piloted by 20 mental health professionals and reviewed by five mental health experts.

### 2.3 Ethical approval and consent to participate

All research methods were carried out following the Helsinki Declaration. The Al Quds University Research Ethical Committee (Ref. No. 347/REC/2023) and the Palestinian Ministry of Health both approved the study. This online survey was intended to be anonymous. The survey started with written information about its purpose and intended use of the data. By completing the questionnaire, participants gave their informed consent to participate in the study.

### 2.4 Data analysis

The data were analyzed with SPSS version 25 (IBM Corp., Chicago, IL, USA). The descriptive analysis for all study variables is reported in the form of frequencies and percentages and chi-square test was performed. Pearson correlation was used to assess the relationship between PTSD symptoms and coping strategies. Furthermore, a multivariate regression analysis was carried out, and the results were given as an adjusted odds ratio (AOR) with a 95% confidence range. The adjusted model included all potential study confounders as well as factors associated with PTSD. A *p*-value of less than 0.05 was considered a significant association.

## 3 Results

In this study, 514 people answered the questionnaire. **Table 1** shows that the majority of participants were women (67.9%), married (73.7%), and aged 30 to 50 (65.6%). The majority (60.1%) had a bachelor’s degree and were employed as psychologists or counselors (66.0%).

**Table 1:** Sociodemographic characteristics of the participants.

		*N*	%
Gender	Male	165	32.1%
	Female	349	67.9%
Age	18–30 years	112	21.8%
	31–40 years	170	33.1%
	41–50 years	167	32.5%
	≥51 years	65	12.6%
Marital status	Single	110	21.4%
	Married	379	73.7%
	Divorced or widowed	25	4.9%
Place of living	City	241	46.9%
	Village	236	45.9%
	Refugee camp	37	7.2%
Monthly income	No income	8	1.6%
	Less than $500	36	7.0%
	$500–$1,000	246	47.9%
	$1,001–$1,500	158	30.7%
	$1,501–$2,500	47	9.1%
	≥$2,501	19	3.7%
Education level	Bachelor degree	309	60.1%
	Master	171	33.3%
	PhD/MD	34	6.6%
Job	Psychotherapists	29	5.6%
	Social worker	102	19.8%
	Psychologist and counselors	339	66.0%
	Psychiatric doctor and nurse	19	3.7%
	Mental health academic staff	25	4.9%

**Table 2** shows that 58.4% of participants had been working in mental health for at least 6 years. Furthermore, 70.4% of participants reported a worsening of their psychological state due to the ongoing Gaza war and Israeli–Palestinian political violence compared to their pre-war state. Furthermore, 43.0% of participants reported treating patients affected by the current Gaza war and Israeli–Palestinian political violence during the first 2 months of the war, while 47.7% reported treating 1 to 15 patients in the first 2 months of the Gaza war. Furthermore, 31.5% reported feeling disabled or unable to care for their patients during the ongoing Gaza war and Israeli–Palestinian political violence. Furthermore, 78.6% of participants said they needed crisis intervention training to treat victims of war and political violence, and the majority (89.5%) said the current Gaza war and Israeli–Palestinian political violence had a negative impact on their mental health.

**Table 2:** Psychological status and history of the participants.

Items		*N*	%
What are your years of experience in the mental health field?	Less than 1 year	51	9.9%
	1–3 years	81	15.7%
	4–6 years	82	16.0%
	7–10 years	94	18.3%
	11–15 years	82	16.0%
	≥16 years	124	24.1%
How long have you been working in your current job?	Less than 1 year	73	14.2%
	1–3 years	135	26.3%
	4–6 years	116	22.6%
	7–10 years	77	15.0%
	11–15 years	48	9.3%
	≥16 years	65	12.6%
Did you receive any training at work about dealing with the victims of crisis, political violence, and war?	Yes	384	74.7%
	No	130	25.3%
Did you receive any training on how to deal with victims of crisis, political violence, and war during your university studies?	Yes	262	51.0%
	No	252	49.0%
Did the current Gaza war and Israeli–Palestinian political violence have negative effects on your psychological state?	Yes	460	89.5%
	No	54	10.5%
How has your psychological state changed as a result of the ongoing Gaza war and Israeli–Palestinian political violence compared to the pre-war period?	Worse now	362	70.4%
	Better now	52	10.1%
	No change	100	19.5%
Did you treat patients suffering from the current Gaza war and Israeli–Palestinian political violence during the first 2 months of the current war?	Yes	221	43.0%
	No	293	57.0%
How many patients did you treat during the current war and the Israeli–Palestinian political violence?	None	125	24.3%
	1–5	148	28.8%
	6–15	97	18.9%
	16–30	65	12.6%
	31–45	14	2.7%
	≥46	65	12.6%
Did you have a feeling of disability or inability to deal with your patients during the current Gaza war and Israeli–Palestinian political violence	Yes	162	31.5%
	No	352	68.5%
Were you exposed to any traumatic events before the current Gaza war?	Yes	113	21.9%
	No	402	78.1%
What types of traumatic or stressful events have you faced in the past?	Death of family members or relatives	55	10.7%
	Political violence	25	4.9%
	Accidents	7	1.4%
	Social stressors such as divorce, or family members problems and physical diseases	16	3.1%
Do you feel that you need training in crisis intervention and treatment of victims of war and political violence?	Yes	404	78.6%
	No	110	21.4%

**Table 3** shows that 38.7% of the sample met the clinical cutoff for PTSD (a score greater than 33), indicating that they are at high risk of receiving a clinical diagnosis of PTSD.

**Table 3:** The prevalence of PTSD symptoms.

PTSD cutoff score	*N*	%
≤33	315	61.3
>33	199	38.7

### 3.1 Associations between respondent characteristics and psychological history and PTSD symptoms

The chi-square test was used to assess the statistical significance of the difference in PTSD categories based on respondent characteristics and other variables. **Table 4** shows that there were significant relationships between PTSD symptoms and age, number of years of experience in the mental health field, participants who reported that the current Gaza war and Israeli–Palestinian political violence had a negative impact on their psychological state, participants who stated that their psychological status was worse now as a result of the ongoing Gaza war and Israeli–Palestinian political violence compared to the pre-war period, and participants who reported feeling disabled or unable to care for their patients during the ongoing Gaza war and Israeli–Palestinian political violence.

**Table 4:** Associations between respondent characteristics and psychological factors and PTSD symptoms.

		PTSD				Chi-square
		≤33		>33		
		*N*	%	*N*	%	*p*-value
Gender	Male	108	65%	57	35%	0.182
	Female	207	59%	142	41%	
Age	18–30 years	64	57%	48	43%	0.042
	31–40 years	94	55%	76	45%	
	41–50 years	110	66%	57	34%	
	≥51 years	47	72%	18	28%	
Marital status	Single	64	58%	46	42%	0.620
	Married	234	62%	145	38%	
	Divorced or widowed	17	68%	8	32%	
Place of living	City	157	65%	84	35%	0.238
	Village	137	58%	99	42%	
	Refugee camp	21	57%	16	43%	
Monthly income	No income	5	63%	3	38%	0.786
	Less than $500	18	50%	18	50%	
	$500–$1,000	153	62%	93	38%	
	$1,001–$1,500	97	61%	61	39%	
	$1,501–$2,500	29	62%	18	38%	
	≥$2,501	13	68%	6	32%	
Education level	Bachelor degree	181	59%	128	41%	0.301
	Master	112	65%	59	35%	
	PhD/MD	22	65%	12	35%	
Jobs	Psychotherapists	17	58.6%	12	41.4%	0.823
	Social worker	59	57.8%	43	42.2%	
	Psychologist and counselors	212	62.5%	127	37.5%	
	Psychiatric doctor and nurse	13	68.4%	6	31.6%	
	Mental health academic staff	14	56.0%	11	44.0%	
What are your years of experience in the mental health field?	Less than 1 year	26	51%	25	49%	0.028
	1–3 years	45	56%	36	44%	
	4–6 years	47	57%	35	43%	
	7–10 years	52	55%	42	45%	
	11–15 years	58	71%	24	29%	
	≥16 years	87	70%	37	30%	
How long have you been working in your current job?	Less than 1 year	39	53%	34	47%	0.290
	1–3 years	85	63%	50	37%	
	4–6 years	66	57%	50	43%	
	7–10 years	50	65%	27	35%	
	11–15 years	35	73%	13	27%	
	≥16 years	40	62%	25	38%	
Did you receive any training at work about dealing with the victims of crisis, political violence, and war?	Yes	241	63%	143	37%	0.238
	No	74	57%	56	43%	
Did you receive any training on how to deal with victims of crisis, political violence, and war during your university studies?	Yes	161	61%	101	39%	0.937
	No	154	61%	98	39%	
Did the current Gaza war and Israeli–Palestinian political violence have negative effects on your psychological state?	Yes	273	59%	187	41%	0.009
	No	42	78%	12	22%	
How has your psychological state changed as a result of the ongoing Gaza war and Israeli–Palestinian political violence compared to the pre-war period?	Worse now	203	56.1%	159	43.9%	0.001
	Better now	38	73.1%	14	26.9%	
	No change	74	74.0%	26	26.0%	
Were you exposed to any traumatic events before the current Gaza war?	Yes	57	51%	55	49%	0.011
	No	258	64%	144	36%	
Did you treat patients suffering from the current Gaza war and Israeli–Palestinian political violence during the first 2 months of the current war?	Yes	137	62%	84	38%	0.775
	No	178	61%	115	39%	
How many patients did you treat during the current Gaza war and the Israeli–Palestinian political violence?	None	81	65%	44	35%	0.307
	1–5	88	59%	60	41%	
	6–15	61	63%	36	37%	
	16–30	43	66%	22	34%	
	31–45	5	36%	9	64%	
	≥46	37	57%	28	43%	
Did you have a feeling of disability or inability to deal with your patients during the current Gaza war and Israeli–Palestinian political violence	Yes	68	42%	94	58%	<0.001
	No	247	70%	105	30%	

Significant at p-value: 0.05.

### 3.2 Associations between coping strategies and PTSD

Pearson correlation was used to assess the relationship between PTSD symptoms and coping strategies. **Table 5** reveals very weak positive correlations between PTSD symptoms and information support (*r* = 0.116, *p* = 0.008), planning (*r* = 0.150, *p* = 0.001), emotional support (*r* = 0.104, *p* = 0.018), humor (*r* = 0.112, *p* = 0.011), and self-distraction (*r* = 0.165, *p* ≤ 0.001). Moreover, the study found weak positive correlations between PTSD symptoms and venting (*r* = 0.278, *p* ≤ 0.001), self-blame (*r* = 0.302, *p* ≤ 0.001), denial (*r* = 0.249, *p* ≤ 0.001), and behavioral disengagement (*r* = 0.277, *p* ≤ 0.001). Finally, the results showed a very weak negative correlation between PTSD symptoms and acceptance (*r* = −0.089,*p* = 0.043).

**Table 5:** Associations between coping strategies and PTSD.

PTSD		
	Pearson correlation	Sig. (two-tailed)
Active coping	0.014	0.753
Information support	0.116^**^	0.008
Positive reframing	0.053	0.234
Planning	0.150^**^	0.001
Emotional support	0.104^*^	0.018
Venting	0.278^**^	<0.001
Humor	0.112^*^	0.011
Acceptance	−0.089^*^	0.043
Religion	0.049	0.264
Self-blame	0.302	<0.001
Self-distraction	0.165^**^	<0.001
Denial	0.249^**^	<0.001
Substance use	0.032	0.466
Behavioral disengagement	0.277^**^	<0.001

Significant at p-value: 0.05.

### 3.3 Multivariate logistic regression for determinants of PTSD

**Table 6** shows the factors that may contribute to the development of PTSD symptoms. Participants who reported feeling disabled or unable to deal with their patients during the ongoing Gaza war and Israeli–Palestinian political violence were nearly three times more likely to develop PTSD than those who did not report feeling this way (OR = 2.992, CI: 1.946–4.601, *p* = 0.001). Furthermore, participants who had experienced traumatic events before the Gaza war were significantly more likely to develop PTSD symptoms than those who had not (OR = 1.757, CI: 1.082–2.854, *p* = 0.023). Participants who used venting (OR = 1.461, CI: 1.242–1.719, *p* ≤ 0.001), self-blame (OR = 1.339, CI: 1.145–1.564, *p* ≤ 0.001), and behavioral disengagement (OR = 1.368, CI: 1.150–1.628, *p* ≤ 0.001) as coping mechanisms were more likely to develop PTSD symptoms compared to those who used other strategies. Finally, the utilization of acceptance (OR = 0.669, CI: 0.540–0.830, *p* = 0.003) and substance use (OR = 0.669, CI: 0.540–0.830, *p* < 0.001) as coping strategies was associated with a reduced likelihood of experiencing symptoms of PTSD.

**Table 6:** Multivariate logistic regression for determinants of PTSD.

				Adjusted analysis	
				95% CI, AOR*	
		Sig.	AOR	Lower	Upper
Were you exposed to any traumatic events before the current Gaza war?	Yes	0.023	1.757	1.082	2.854
	No	Reference			
Did you have a feeling of disability or inability to deal with your patients during the current Gaza war and Israeli–Palestinian political violence	Yes	<0.001	2.992	1.946	4.601
	No	Reference			
Venting		<0.001	1.461	1.242	1.719
Acceptance		0.003	0.808	0.701	0.930
Self-blame		<0.001	1.339	1.145	1.564
Substance use		<0.001	0.669	0.540	0.830
Behavioral disengagement		<0.001	1.368	1.150	1.628

Multivariate logistic regression model: Adjusted for gender, age, living, monthly income, education level, job, years of experience in the mental health field, years of work in your current job, whether you received any training in your workplace about dealing with the victims of crisis and political violence and war, whether you received any training during your university study about dealing with the victims of crisis, political violence, and war; whether you were exposed to any traumatic events before the Gaza war, whether your psychological state changes during the war and political violence in comparison to before the war, whether you treated patients who suffer from the Gaza war and political violence in the West Bank, the number of patients they treated, whether you have a feeling of disability or inability to deal with your patients during the Gaza war, active coping, information support, positive reframing, planning, emotional support, venting, humor, acceptance, religion, self-blame, self-distraction, denial, substance use, and behavioral disengagement.

## 4 Discussion

The mental health of mental health professionals in Palestine is a pressing issue since they are responsible for providing long-term support to the Palestinian population. The current study found that more than one-third of Palestinian mental health professionals (38.7%) are at high risk of receiving a clinical diagnosis of PTSD. This finding is considered high when compared to other studies in the literature review. For example, the prevalence of PTSD among healthcare professionals ranged between 0% and 30% (30, 31), whereas it varied between 0% and 17% among mental health professionals (32, 33). Furthermore, a study in Botswana by Olashore et al. showed that 18.4% of mental health professionals experienced PTSD (34). Vicic and Motta found that the percentage of mental health professionals who experienced secondary trauma ranged between 23% and 27% (35). In addition, Jacobowitz reported that 9%–10% of mental health professionals had PTSD (36). Variations in prevalence between studies could be attributed to differences in methodology approaches, cultural influences on reporting, and transparency in scale questions answered (37). Furthermore, the severe and ongoing Israeli–Palestinian conflict, as well as recurring wars, may cause enormous suffering and significant harm to the mental health of Palestinian mental health workers. According to research, the prevalence of PTSD in conflict and war zones increased as the conflict lasted longer (38). Furthermore, it argues that the term “posttraumatic stress disorder” originated in Western culture and specifically refers to the traumatic experiences that soldiers face in combat. Their concerns are unlikely to originate from a sense of immediate physical threat or danger. However, flashbacks and nightmares feel very real to anyone who has experienced them; they maintain a sense of current threat, which is what maintains PTSD. In contrast, for a Palestinian whose home has been specifically targeted, the threat of another attack is real. As a result, the concept of a “post” phase may be inappropriate because the trauma is repeated, persistent, and ongoing (39). Therefore, further research is needed to assess the fundamental mechanisms and theories behind the emergence of mental health problems associated with persistent and long-lasting trauma in the Palestinian population (39).

Mental health professionals who exhibit PTSD symptoms have a higher rate of absenteeism, turnover, and ineffective treatment of patients (40). Furthermore, if PTSD symptoms are not treated, they may become emotionally detached, reducing their ability to provide responsive, empathetic, and caring support to clients. As a result, it is critical to consider the possibility that mental health professionals have PTSD and to provide them with psychological support and interventions at work.

The current study further found that prior exposure to traumatic events could predict the development of PTSD, which is consistent with previous studies (41–43). According to Adams and Riggs’ research in the United States, 38.7% of psychologists had experienced personal trauma (44). A study by Breslau et al. revealed that repeated trauma exposure has more negative consequences than a single exposure (45). Emotional distress is experienced by mental health professionals immediately after a traumatic event and up to a year later (8) as a result of widespread poverty, hunger, harm, and economic and societal downturns, as well as emotions of loss, fear, anguish, and sadness (1, 46). Thabet et al. found that major depression and PTSD often coexist and can exacerbate each other’s symptoms (47). Lindsay’s study of Palestinian mental health professionals found that experiencing war as a victim or survivor increased stress and anger in mental health professionals, which contradicted their professional values (48).

Furthermore, the current study found that mental health professionals who felt incapable of caring for their patients as a result of the ongoing Gaza war and Israeli–Palestinian political violence were at a high risk of developing PTSD. This finding is consistent with previous research (9, 11, 49). Eidelson et al. reported that half of the therapists felt unprepared for their wartime jobs (46). According to Batten and Orsillo, some therapists believed their efficacy had declined, and many were tired of hearing about the same traumatic event repeatedly (49). On the other hand, Veronese et al. revealed that Palestinian mental health professionals experienced moderate psychological distress while maintaining a strong sense of coherence (50). However, the current level of violence is unprecedented in previous wars or periods of political violence, which may explain some of the study’s findings. This war may also have an impact on how mental health professionals perceive their own compromised competence. Furthermore, providing psychological interventions during large-scale traumatic events may cause distress among mental health workers (51). For example, following 9/11, the vast majority of aid workers in the United States stated that they felt helpless due to the challenges posed by the large number of people attempting to manage severe suffering (46).

In addition, the current study found that participants who used venting, self-blame, and behavioral disengagement as coping mechanisms were more likely to develop PTSD symptoms than those who used other coping strategies. Carver classified behavioral disengagement, venting, self-distraction, self-blame, and substance abuse as maladaptive strategies (52). Similarly, other studies showed that people with PTSD frequently engage in ineffective coping strategies such as venting, behavioral disengagement, and self-blame (15, 50, 52). The use of these coping strategies causes increased stress responses (53). For example, Viana Machado et al. revealed that self-blame is strongly associated with posttraumatic stress symptoms because people who have strong feelings of responsibility and frequently engage in self-criticism may have a maladaptive coping strategy (50). Other researchers argue that because of their education and experience, health professionals may engage in a variety of maladaptive coping strategies to function for the benefit of others (54).

Moreover, in this study, participants who used acceptance and substance use as coping mechanisms were less likely to develop PTSD symptoms. One study found that using acceptance as a coping mechanism reduced PTSD symptoms, while alcohol increased stress responses (55). Woodward et al. found that those with higher levels of emotional nonacceptance experienced the most severe PTSD symptoms (55). Sinnott et al. showed that acceptance had the greatest potential for reducing PTSD by giving people a sense of control over their own lives (56). When confronted with situations over which they believe they have little control, people prefer to accept rather than intervene directly (57). Active acceptance allows a person to redirect their energy away from a situation in which additional effort would be futile and instead focus it on other, more effective actions (58). Other studies have identified acceptance as a maladaptive coping mechanism. Kearns et al. showed that increased use of acceptance coping strategies was linked to a higher risk of developing severe PTSD symptoms. Acceptance strategies in stressful situations may indicate learned helplessness, leading to worsening PTSD symptoms (59). However, Sinnott’s research revealed no link between acceptance and PTSD symptoms (56).

Finally, the current study found that substance use increases the risk of developing PTSD symptoms. Other studies found a link between substance use and PTSD (60, 61). Individuals suffering from PTSD may turn to substance abuse to alleviate the recurring distressing memories and intrusive symptoms associated with the disorder, such as negative thoughts, flashbacks, or pain. This is related to the self-medication hypothesis because people use substances to alleviate the psychological stress caused by PTSD symptoms (62). While this coping mechanism may temporarily relieve trauma symptoms, it has the potential to result in long-term PTSD (63). Additionally, Chilcoat and Breslau found that people with PTSD are more likely to develop a substance use disorder (45). Roberts et al. (2016) proposed that, due to their education and experience, health professionals may engage in a variety of maladaptive coping strategies to function for the wellbeing of others (54).

This research had limitations. Convenience sampling and cross-sectional designs impose limitations on the capacity to establish causal relationships. Additionally, there is a possibility of reporting bias due to the utilization of a self-reported questionnaire. Given that recruitment was carried out via platforms such as Google Docs and WhatsApp, it is likely that mental health professionals currently employed in the affected area, including the Gaza Strip, do not have access to or the opportunity to make use of this technology. As a result, this circumstance may affect the sample’s representativeness. Notwithstanding these limitations, the present study’s results provide insight into the psychological wellbeing of mental health professionals residing in regions affected by conflict. This study makes a significant contribution to the existing literature by being the first to evaluate PTSD in mental health professionals from Palestine during periods of armed conflict and political violence.

### 4.1 Implications of the study

As a result, professionals must develop effective coping strategies early in their careers to reduce the risk of developing PTSD. Furthermore, mental health professionals with PTSD should learn these techniques to aid in their successful reintegration. However, teaching these skills to others is difficult. To fully internalize and demonstrate the acquired skills, the individual must actively engage in using these techniques and discussing personal experiences, as well as examples from other people’s lives (52). Thus, supervision may be required for mental health professionals who use immature defense mechanisms, especially when dealing with countertransference reactions. Furthermore, training sessions or seminars can help mental health practitioners understand their typical defense style and how it affects them and their ability to provide therapy (44).

To improve the competence and professional wellbeing of Palestinian mental health professionals, Palestinian universities, and mental health institutions should establish comprehensive and long-term courses and training programs. These initiatives seek to empower professionals while instilling a sense of autonomy and self-esteem. Furthermore, mental health professionals should receive psychological support, treatment, and supervision at the workplace to overcome their negative emotions. We need more research to understand how war and political violence affect healthcare workers on the job, particularly when they become victims rather than caregivers. More qualitative and quantitative research is required to fully understand coping strategies for dealing with traumatic events.

## 5 Conclusions

The study found that PTSD symptoms are common among Palestinian mental health professionals. Feeling disabled or unable to care for patients during a political conflict or war, as well as a history of trauma, increases the risk of developing PTSD symptoms. In addition, using venting, self-blame, and behavioral disengagement as coping mechanisms increases the likelihood of developing PTSD symptoms. Additionally, using acceptance and substance use as coping mechanisms reduces the risk of developing PTSD symptoms. Consequently, mental health professionals need immediate assistance in enhancing their mental wellbeing through supervision, psychotherapy, and comprehensive and continuous training.

## Data availability statement

The original contributions presented in the study are included in the article/supplementary material. Further inquiries can be directed to the corresponding author.

## Ethics statement

The studies involving humans were approved by Al Quds University Research Ethical Committee (Ref No: 347/REC/2023). The studies were conducted in accordance with the local legislation and institutional requirements. The participants provided their written informed consent to participate in this study.

## Author contributions

MA: Conceptualization, Data curation, Formal analysis, Investigation, Methodology, Project administration, Software, Supervision, Validation, Writing – original draft, Writing – review & editing. MAT: Conceptualization, Investigation, Validation, Writing – review and editing. LF: Conceptualization, Investigation, Validation, Writing – review & editing.
